# Absorption Correction for 3D Elemental Distributions of Dental Composite Materials Using Laboratory Confocal Micro-X-ray Fluorescence Spectroscopy

**DOI:** 10.1021/acs.analchem.4c00116

**Published:** 2024-05-17

**Authors:** Leona J. Bauer, Frank Wieder, Vinh Truong, Frank Förste, Yannick Wagener, Adrian Jonas, Sebastian Praetz, Christopher Schlesiger, Andreas Kupsch, Bernd R. Müller, Birgit Kanngießer, Paul Zaslansky, Ioanna Mantouvalou

**Affiliations:** † Institute for Optics and Atomic Physics, Technical University of Berlin, Hardenbergstr. 36, 10623 Berlin, Germany; ‡ Berlin Laboratory for innovative X-ray technologies—BLiX, Berlin 10623, Germany; § Helmholtz-Zentrum Berlin, Albert-Einstein-Str. 15, 12489 Berlin, Germany; ∥ Bundesanstalt für Materialforschung und -prüfung (BAM), Unter den Eichen 87, 12205 Berlin, Germany; ⊥ Physikalisch-Technische Bundesanstalt, Abbestraße 2-12, 10587 Berlin, Germany; # Department for Operative, Preventive and Pediatric Dentistry, Charité—Universitätsmedizin Berlin, Aßmannshauser Str. 4-6, 14197 Berlin, Germany

## Abstract

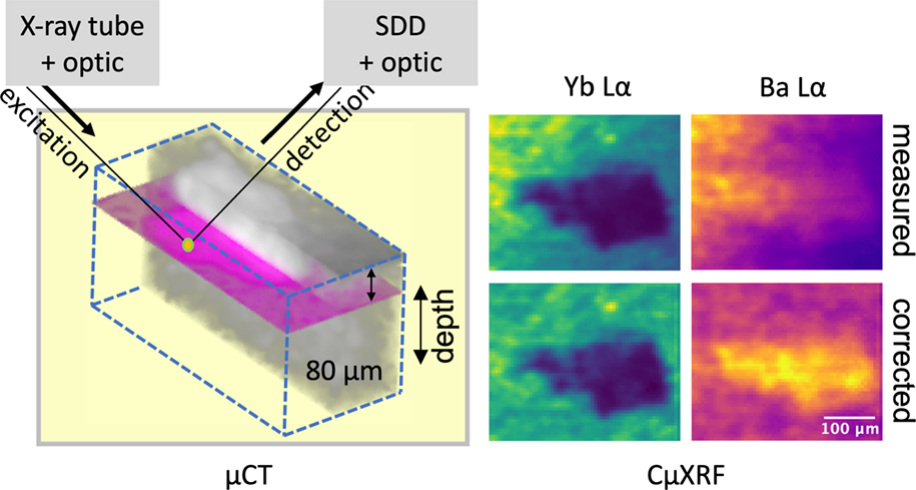

Confocal micro-X-ray fluorescence (micro-XRF) spectroscopy facilitates three-dimensional (3D) elemental imaging of heterogeneous samples in the micrometer range. Laboratory setups using X-ray tube excitation render the method accessible for diverse research fields but interpretation of results and quantification remain challenging. The attenuation of X-rays in composites depends on the photon energy as well as on the composition and density of the material. For confocal micro-XRF, attenuation severely impacts elemental distribution information, as the signal from deeper layers is distorted by superficial layers. Absorption correction and quantification of fluorescence measurements in heterogeneous composite samples have so far not been reported. Here, an absorption correction approach for confocal micro-XRF combining density information from microcomputed tomography (micro-CT) data with laboratory X-ray absorption spectroscopy (XAS) and synchrotron transmission measurements is presented. The energy dependency of the probing volume is considered during the correction. The methodology is demonstrated on a model composite sample consisting of a bovine tooth with a clinically used restoration material.

## Introduction

**Figure 1:**
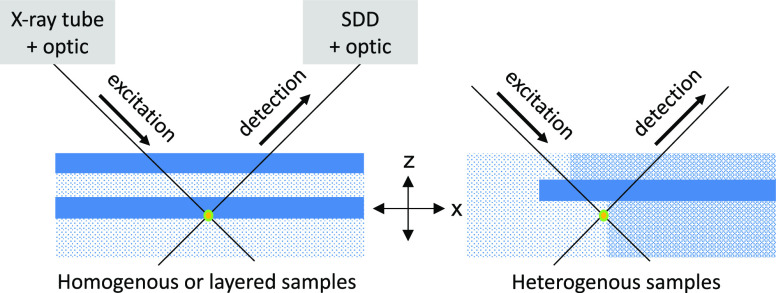
Schematic view of different sample types in relation to the confocal micro-XRF geometry. Different patterns depict areas in the samples with different composition and density. The intersection of excitation and detection path marks the probing volume from which information is derived.

Confocal micro-X-ray fluorescence (confocal micro-XRF) spectroscopy is a method that nondestructively investigates three-dimensional (3D) elemental distributions of a large variety of sample classes.^1−6^ Laboratory setups using conventional microfocus X-ray tubes and energy-dispersive detectors make it possible to create 3D spectroscopic data of the elemental composition of large-scale objects through the localized excitation of atoms and the resulting subsequent fluorescence emission. To measure the 3D distribution, the sample is scanned through the probing volume, which is formed by an overlap of the foci of the two polycapillary optics of the spectrometer—one focusing the X-ray radiation from the source onto the sample, the other collecting the scattered and emitted fluorescence radiation directing them to the silicon drift detector (SDD, Figure 1). Because the focusing principle of polycapillary optics is based on energy-dependent total reflection, the size and sensitivity of the probing volume are also energy-dependent.^7^ Additionally, the 3D measurements are subject to material and energy-dependent absorption effects where both the incoming excitation radiation and the emitted scattered and fluorescence radiation are attenuated. On the one hand, these effects complicate the analysis of the measured intensity signals per depth, requiring absorption correction. On the other hand, the attenuation of the measured spectra represents information that can be used to extract the quantitative composition of the sample.

For homogeneous bulk samples with a homogeneous matrix and density approaches to correct the measured intensities have already been reported.^8,9^ Using monochromatic synchrotron excitation, quantification of elemental concentrations becomes feasible.^10,11^ Also, for layered samples (Figure 1 left), there are approaches to correct the confocal micro-XRF data for absorption to quantify the layer thickness and composition of the samples in-depth.^12−14^ For heterogeneous composite samples, though, where the absorption properties have to be considered in the excitation and detection paths (Figure 1, right), correction of the measured fluorescence intensities and the quantification of elemental distributions are still challenging. Precise knowledge of the structure, density, and matrix of the different parts of the composite sample is necessary for an absorption correction and, ultimately, for a full 3D quantification of the elemental distribution.

A previous study showed that micro-XRF and confocal micro-XRF can be utilized to qualitatively image the diffusion of elements from commonly used dental filling materials leaching into the tooth tissue.^15^ For a detailed analysis of the transition zones formed between the materials (dentine, filling), absorption and energy effects have to be corrected. Here, an absorption correction procedure for laboratory confocal micro-XRF measurements based on a voxel-wise correction using the Lambert–Beer law previously introduced by Mantouvalou et al.^8^ is presented. The geometry of the setup and the sample are considered to calculate the attenuation of the exciting and fluorescence radiation in the different materials of the composite sample in order to correct the measured fluorescence peak intensities in each voxel. The absorption coefficients of the materials are derived by combining information provided from laboratory X-ray absorption spectroscopy (XAS) measurements and synchrotron transmission measurements. Laboratory micro-X-ray computed tomography (micro-CT) data of the sample is used to derive a 3D density model and use this density information for absorption correction. In the process, the energy-dependent probing volume size is accounted for.

The work presented here demonstrates on the one hand the feasibility to derive absorption corrected elemental distributions of composite materials with different compositions and densities. On the other hand, the absolute composition of the materials is derived by combining different X-ray techniques. While the latter necessitates resource hungry experiments and analysis, the former is already applicable, if a good assumption for the dark matrix, composition, and density of the materials is given. This can in the simplest case be derived through an initial assumption, or also be measured with other analytical techniques such as mass spectrometry, or chemical analysis.

## Experimental Section

### Materials

**Figure 2:**
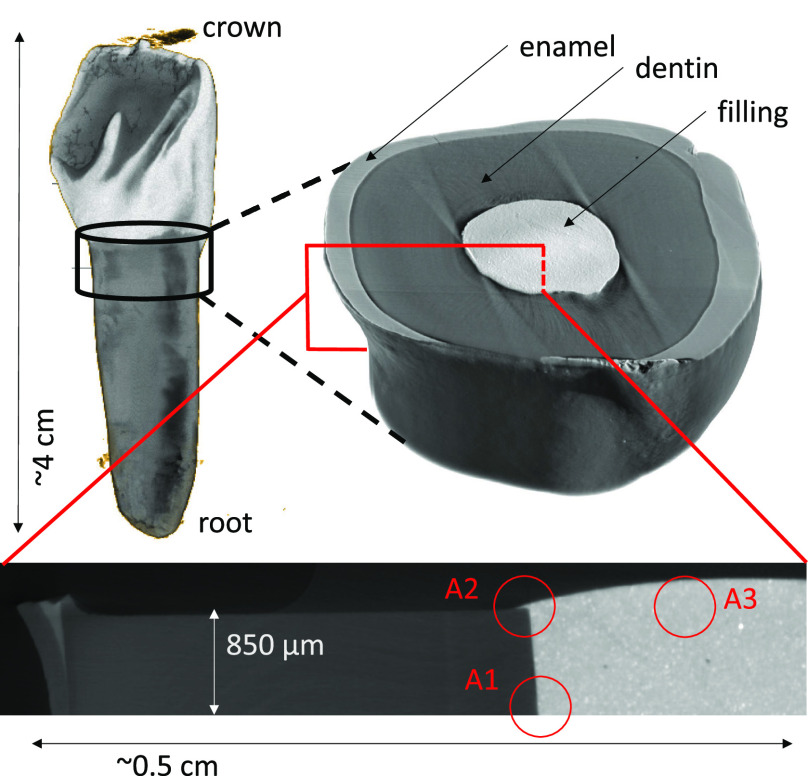
Top-left: Reconstructed micro-CT volume of a full bovine tooth. Top-right: 3 mm thick cross section of the bovine tooth with an SDR flow+ filling in the root canal, which was cut for further measurements to a thickness of ∼850 μm (sample T1). Bottom: slice of half of the cross section indicating the measured areas A1–3.

A 850 μm thick slice of a bovine tooth (Figure 2, bottom) filled with a commonly used light curing dental composite (SDR flow+ composite universal from Dentsply Sirona, USA) in the pulp chamber is used as a composite sample (T1). The composite filling material contains heavy elements, including barium, ytterbium, and strontium.^16^

An additional bovine tooth and samples of the filling material are used separately to create samples for the measurement of absorption cross sections (T2 (thickness of 144 μm), SDR-thin (123 μm), SDR-thick (413 μm)). Further information on the sample preparation is provided in the Supporting Information S1.1. The density of dry bovine dentine and SDR flow+ are measured to be (2.025 ± 0.075) g/cm^3^ and (2.15 ± 0.15) g/cm^3^, respectively. We measured the volume of bovine dentine and SDR flow samples and weighed them to determine the density. For the dentine samples, we used micro-CT measurements (10 μm voxel size) to determine the volume with higher accuracy, as the samples could not be prepared as perfect cubic shapes.

### Methods

#### Micro-X-ray Fluorescence and Confocal Micro-X-ray Fluorescence

For micro-XRF and confocal micro-XRF measurements, a laboratory spectrometer equipped with two polycapillary optics (Helmut Fischer GmbH, Berlin, Germany), a SDD detector (Bruker Nano GmbH, Berlin, Germany), and a 30 W microfocus molybdenum X-ray tube (rtw, Neuenhagen, Germany) is used.^17^ To change between micro-XRF and confocal micro-XRF geometries, the SDD is moved between two fixed ports. Realignment is not necessary because of the high mechanical stability of the spectrometer head. Due to ambient pressure and the transmission of the X-ray optics and the detector window, the spectrometer enables elemental imaging for elements with atomic number >14 and >19 for micro-XRF and confocal micro-XRF, respectively.

Measurements are performed at 30 W (50 kV and 600 μA) and 9 W (30 kV and 300 μA). For micro-XRF measurements, 10 μm step size and 20 s real-time are used. The size of the confocal probing volume, which is formed by two polycapillary optics, is experimentally determined using thin metal foils. For the experiments presented here, it ranges from a full width at half of the maximum (FWHM) of 47 μm for Ca Kα to 15 μm for Sr Kα (see Supporting Information S1.2).

Three confocal micro-XRF measurements on the sample are presented in Figure 2 (bottom). Virtual 2D slices measured in *xz*- and *xy*-direction (see Figure 1) at three different areas are shown. Area 1 (A1, Figure 2, bottom) represents an interface between dentine and filling material without any overlap of the two materials. Here, two virtual *xz*-slices are measured at approximately the same position with the sample rotated manually by 180°. Consequently, the orientation of filling and dentine relative to the excitation and detection path are exchanged. In area 2 (A2), an interface is measured where the filling material partially overlaps the dentine. Area 3 (A3) is located inside the filling material where a micron-sized inclusion with a different density and composition than the filling is identified by micro-CT. Here, both a 3D volume with low resolution and a virtual cross section at a depth of 80 μm below the surface are measured. The 3D volume is used to match the confocal micro-XRF data set with the micro-CT and to be able to determine the absorption properties. The confocal micro-XRF measurement parameters are shown in Table 1.

**Table 1:** Experimental Parameters and Settings of the X-ray Tube during Confocal Micro-XRF Measurements at Areas A1, A2, and A3 of Sample T1

area	measurement	step size in *x*, *y,* and *z*	real-time	X-ray tube settings
A1	2 *xz* slices	6 μm	130 s	9 W, 30 kV
A2	*xz* slice	20 μm	203 s	30 W, 50 kV
A3	*xyz* volume	18 μm	60 s	30 W, 50 kV
A3	*xy* slice	6 μm	30 s	30 W, 50 kV

The detected spectra at each measuring point are normalized to the lifetime derived from the zero peak in each spectrum and then deconvolved using a custom-made in-house Python code *specfit*.^12^ The 2D data sets are visualized using the matplotlib Python package (3.7) and ImageJ (2.1.0).

#### Micro-X-ray Computed Tomography

The untreated and treated bovine teeth (Figure 2) are measured by a laboratory micro-CT microscope (Xradia 620 Versa, ZEISS) equipped with a tungsten X-ray tube producing a cone beam. A flat panel X-ray detector is used to measure the transmitted radiation. The excitation is set to 90 kV and 0.11 mA. To reduce beam hardening caused by polychromatic excitation, a filter (LE5) is used. 2400 and 3000 projections are collected, with an exposure time of 2 s. The effective pixel sizes are 24 and 6.2 μm for teeth without and with filling, respectively. micro-CT reconstruction is performed using Zeiss Software.

#### X-ray Transmission Measurement

X-ray transmission measurements at several energies (17.5 and 20 keV to 32 keV in 2 keV steps) are performed using the synchrotron X-ray refraction radiography^18,19^ setup at BAM*line*^20,21^ at the synchrotron radiation facility BESSY II (Berlin, Germany).^22^ A fluorescence screen, a system of lenses, and a pco.1600 CCD camera (PCO AG, Kelheim, Germany) are used as detection system.^21^

Measurements are performed on T1 on the tooth tissue and the filling region, with an additional measurement performed off the sample at each energy setting. The product of the linear mass absorption coefficient μ(*E*) and the thickness of the sample *d* can be calculated using the Lambert–Beer relation:
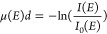
1where *I*(*E*) is the intensity measured on the sample and *I*_0_(*E*) off the sample. With the known thickness *d*, the linear mass absorption coefficient μ(*E*) for the two materials at different energies can be calculated.

Additional transmission measurements of T2 and SDR-thin are performed at the four-crystal monochromator (FCM) beamline for radiometry at BESSY II^23^ within the energy range from 3 to 10 keV. The transmission is measured using a thin photodiode placed in front of the sample and a calibrated photodiode placed behind the sample. The thin diode served as an online *I*_0_(*E*) monitor for the transmission and is counter-calibrated in a previous measurement where the sample is removed from the beam. This allowed for simultaneous measurement of *I*_0_(*E*) and *I*(*E*)*.* The Lambert–Beer equation (eq 1) yields μ(*E*)*d*.

#### X-ray Absorption Spectroscopy

The absorption of the two SDR flow+ disks is further measured by XAS using a laboratory von Hamos-based spectrometer.^24^ The spectrometer is equipped with a molybdenum X-ray tube optimized to reach 30 W at 15 kV (rtw, Neuenhagen, Germany) and has a pixelated X-ray detector (Dectris Eiger2 R 500 K). A highly annealed pyrolytic graphite (HAPG) optic is used as the dispersive element, which, in combination with the 2D detector, provides a spectral bandwidth of ∼300 to ∼500 eV. Four different energy ranges at the Ca K, Ba L3 and L2, Yb L3, and Sr K edges are measured using an exposure time of 1800 s and 5 to 10 images each, depending on the signal-to-noise ratios. For each setting, a reference measurement is performed without the sample to derive μ(*E*)*d* according to eq 1.

#### Reference-free X-ray Fluorescence

To gain access to the composition of the light elements smaller *Z* = 14, reference-free XRF quantification is performed on the plane grating monochromator (PGM) beamline in the PTB laboratory at BESSY II^25^ in the soft X-ray range. Reference-free quantification is based on calibrated instrumentation and the knowledge of all experimental parameters and fundamental parameters (FP).^26^ The beamline operates between 80 and 1860 eV. For the quantification of C, N, O, and F, an excitation energy of 760 eV is used. For the quantification of Na, Mg, and Al, the excitation energy is set to 1622 eV. The measured fluorescence intensities are compared with a forward calculation using the Sherman equation, and the mass fractions in weight-percent (wt %) of the elements in question are adjusted using a fit routine.^27^ In the fitting algorithm, the mass fractions of all heavier elements are fixed.

## Data Analysis

A voxel-wise correction is used as introduced by Mantouvalou et al.^8^ of the measured net peak intensity *I*_meas,*j*_ of the fluorescence line (*j*) of an element at every respective voxel (*x,y,z*) based on the Lambert–Beer eq 1 to calculate the absorption corrected intensity values *I*_corr,*j*_ by
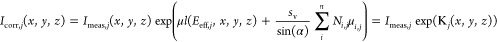
2where the first and the second terms in the exponent describe the attenuation in the excitation and detection path, respectively. In the excitation path, the attenuated spectrum in each depth is calculated, and the product of the effective absorption coefficient and depth μ*l* of each voxel at positions *x, y,* and *z* is determined. The determination of the μ*l* term in the excitation path is described in further detail in Supporting Information S2.1. In the detection path, a linear combination of the absorption coefficients of the different materials μ_*i,j*_ is used.

From the size of the voxels *s*_v_, detection angle α, and the number of voxels in the detection path *N*_*i,j*_*,* the detection path length inside the materials is calculated. The two terms of the exponent, which describe the attenuation in both paths, are summarized in the correction term Κ*_j_(x,y,z).* This equation can be solved for each of the fluorescence lines of the relevant elements individually if the mass absorption coefficient μ(*E*) of the entire sample is known. The structure of the sample, in particular the number of voxels in the excitation path and detection paths within the dentine or filling material, are derived from the segmented micro-CT data, aligned with the confocal micro-XRF data. Segmentation is performed using simple thresholding methods and registration is performed with a self-written Python code using template matching (see Supporting Information S2.2).

The energy-dependent linear absorption coefficients for the SDR flow+ filling and bovine dentine are calculated from transmission and absorption measurements (see above) as well as from theoretical calculations using the density and the chemical composition of the materials.

The correction procedure requires the correction term *K*_*j*_ for each voxel of the measured area for the fluorescence line of interest. The intensity signal in each voxel is corrected using eq 2 and an array containing the previously determined correction terms. Every voxel is treated with the assumption that the probing volume is a point, and that excitation and detection paths are infinitesimal thin lines. To consider the size of the probing volume, the information of the FWHM derived from depth scans of thin foils (see above and Supporting Information S1) is used. Using an exponential fit to the measured FWHM’s, a value for the fluorescence line energy of all elements is calculated. Example 2D Gaussian distributions are shown in Figure 5 (right) for Ca Kα, Yb Lα, and Sr Kα in the micro-CT virtual slice of the interface between dentine and filling at A1. For the Ca Kα fluorescence, the size of the 2σ region of the probing volume spans 13 voxels in diameter. Thus, it cannot be neglected in the evaluation, especially at the interfaces. Using the information from the Gaussian distribution, it is possible to sum up the weighted correction terms of the voxels in a circular area (radius = 2σ) around each voxel both in the *xz* direction and perpendicular to it. No interpolation is performed here, and only voxels that are within the radius are considered. This way, by using the resulting optimized array of correction terms, the energy-dependent size of the probing volume is considered for the absorption correction.

## Results

### Linear Mass Absorption Coefficient and Elemental Composition

**Figure 3:**
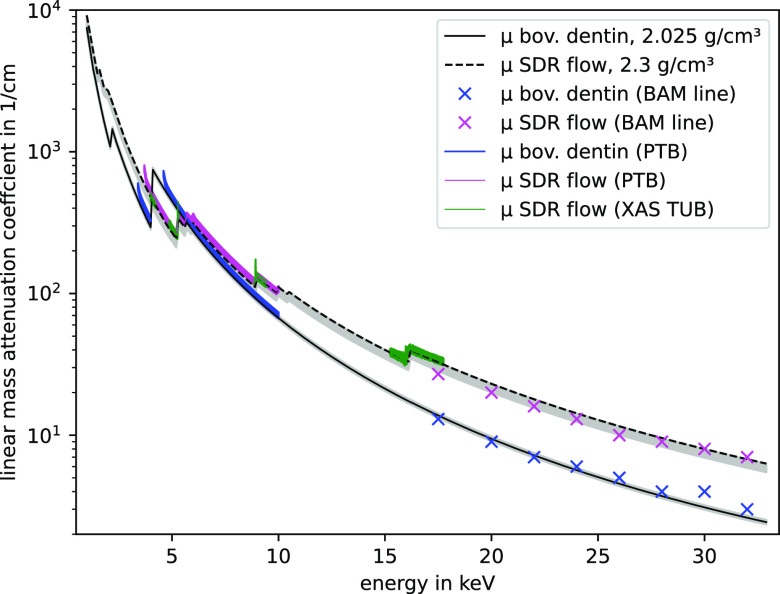
Linear mass absorption coefficients of SDR flow+ (magenta, green, and black) and bovine dentine (blue and black). Solid lines and cross marker points are derived from transmission measurements at BAM and PTB beamlines at BESSY II and laboratory XAS measurements at the TU Berlin. The gray area shows the range of the calculation assuming a density uncertainty of ±0.15 g/cm^3^ (SDR flow) and ±0.075 g/cm^3^ (bov. dentine).

The measured and calculated linear mass absorption coefficients for the SDR flow+ filling and bovine dentine are shown in Figure 3. The colored solid lines and cross marker points show the measured values, the solid and dotted black lines show the calculated values, and the gray area shows the range of the calculated values identified by the density uncertainty. The linear mass absorption coefficient of the materials is significantly different, especially for energies below 5.5 keV where multiple absorption edges are found.

For the calculation of the determined absorption coefficient, a mean composition of the two components—filling material and bovine dentine—must be assumed. As this assumption is ambiguous, micro-XRF information and FP quantification of the soft-X-ray measurement at PTB can be used as a validation. Starting values for the composition of the filling and the dentine tissue from the literature^16,28,29^ allow to calculate the linear mass absorption coefficient and compare it with the measured values derived from XAS and transmission measurements. In addition, the setup parameters of the laboratory spectrometer and composition values are used to forward calculate micro-XRF intensities using the Sherman equation. These are then compared with the measured fluorescence intensities. The elemental composition is adapted and compared again with the information derived from reference-free XRF, micro-XRF, and XAS measurements until the deviations of measured and calculated values are minimized. Table 2 shows the final elemental composition of the filling and the dentine tissue that was used for calculating the linear mass absorption coefficients in wt % or mg/kg (for values less than 1 wt %) and the corresponding uncertainties.

**Table 2:** Quantitative Composition of SDR Flow+ (A) and Bovine Dentine (B) Derived from Measured Linear Absorption Coefficient Values Combined with FP Quantification of Reference-free XRF Measurements as well as Validation Calculations Using Micro-XRF Intensities^a^

A														
H (wt %)	C* (wt %)	N* (wt %)	O* (wt %)	F* (wt %)	Na* (mg/kg)	Al*(wt %)	Si (wt %)	Cl(mg/kg)	K(mg/kg)	Ca(mg/kg)	Ti(mg/kg)	Sr (wt %)	Ba (wt %)	Yb (wt %)
3	36	2.9	31	3.7	1400	3.8	3.5	3000	400	900	1500	3.5	12.0	6.0
2–6	±4	±0.4	±4	±0.5	±300	±0.5	±1.2	±900	±200	±300	±500	±0.4	±1.2	±0.6

B															
H (wt %)	C* (wt %)	N* (wt %)	O* (wt %)	F* (mg/kg)	Na* (wt %)	Mg* (wt %)	Al* (mg/kg)	P (wt %)	S (mg/kg)	Cl (mg/kg)	Ca (wt %)	Cu (mg/kg)	Zn (mg/kg)	Sr (mg/kg)	Ba (mg/kg)
2.1	16	5	35	800	1.2	1.2	2300	11.5	4000	500	26.8	30	200	270	210
(2.0–2.2)	(11–17)	(3–5)	(33–40)	±200	±0.2	±0.2	±300	±3	±1200	±200	±2.7	±10	±60	±80	±60

a Elements marked with a * are validated with reference-free XRF in the soft X-ray range only, the values for H are based on stoichiometric estimation. Relative uncertainties, ranging from 10 to 30%, are given in the rows below the values. Ranges are given for H, C, N, O wt % that reflect the biodiversity of the dentine samples.

The uncertainties of the reference-free XRF quantification originate from the uncertainties of the FP parameters. The uncertainty of the K fluorescence yield is the largest and contributes more than half of the uncertainty. For micro-XRF measurements in the lab, the calibration of the setup is an additional relevant uncertainty factor. Micro-XRF quantification uncertainty ranges from 10% for main components to 30% for the lower-Z elements (P to K) and trace elements. The forward calculated and measured micro-XRF intensities are found in the Supporting Information S3.1. In the case of the dentine composition, instead of the uncertainties of the dark matrix elements H, C, N, and O, a range is given that takes into account the biodiversity of the tooth samples, spanned by stoichiometric calculations based on the structural formulas for the mineral part of dentine found in the literature^29^ and the deviation of different teeth measured by soft X-ray measurements and (confocal) micro-XRF.

### Absorption Corrected Elemental Distributions

**Figure 4:**
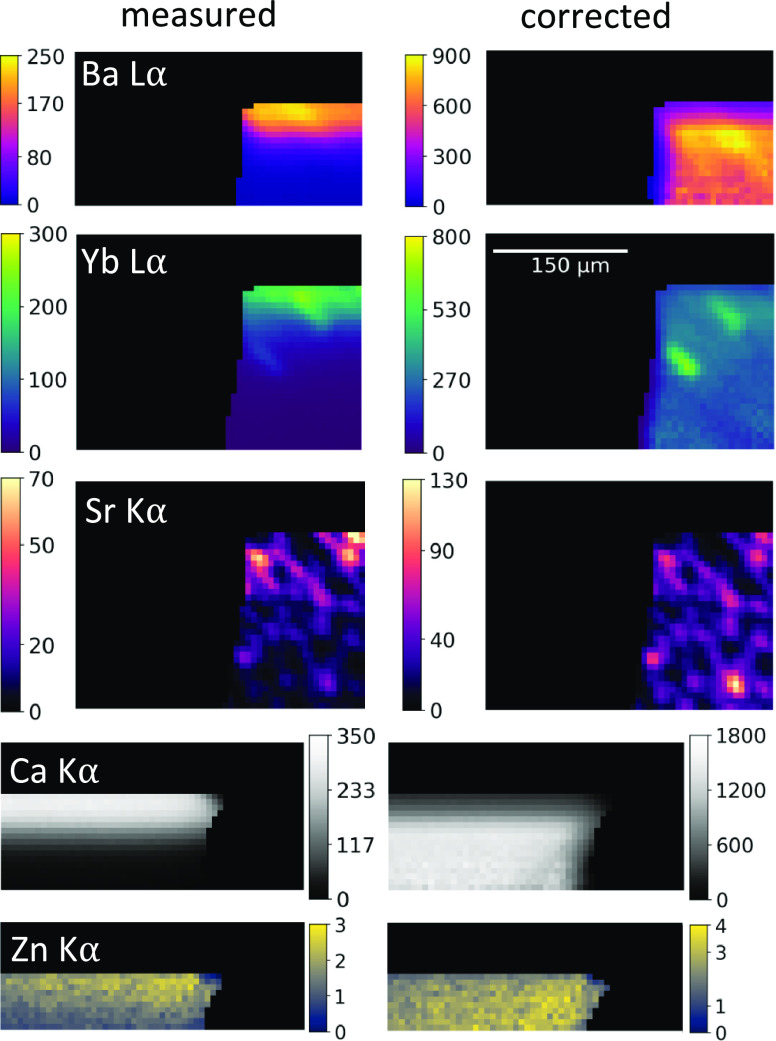
Measured and corrected elemental distributions of Ba, Yb, Sr, Ca, and Zn at A1 first orientation. Color scales show the intensity (cps) of the fluorescence.

To demonstrate the absorption correction procedure, measured and absorption-corrected elemental distributions of the different investigated areas on the tooth sample T1 (A1, A2, and A3) are shown. Distributions of Ba Lα, Yb Lα, and Sr Kα in the filling and Ca Kα and Zn Kα in the dentine are presented in Figure 4 for one sample orientation. The other orientation is depicted in the Supporting Information S3.2. The left column shows the measured net peak fluorescence intensities in counts per second (cps), and the right column shows absorption corrected intensities considering the energy-dependent size of the probing volume. The energy dependency of the absorption effect is clearly visible in the measured elemental distributions: while for Ba Lα and Ca Kα, the intensity decreases within 10 voxels to 10%, the absorption is barely visible for the Sr Kα distribution throughout the full measurement of 31 voxels. After correction, the elemental distributions within both the dentine and the filling are homogeneous into the depth, with the filling material exhibiting inclusions of approximately 20 μm × 30 μm size. During the absorption correction procedure, intensities are corrected for all voxels, where the statistical error of the number of counts in the fluorescence signal is lower than 10%. This leads to differences in the depth from which information can be interpreted reliably, depending on the energy of the observed elemental line energy. From the absorption-corrected elemental distributions of the two measurements at A1, the mean net peak fluorescence intensities of a square within the filling and the dentine for both orientations are determined. For the Yb Lα distributions, regions without any inclusions are used. The results are shown in Table 3. The mean net peak intensities show that the absorption correction procedure delivers the same corrected intensity values with deviations less than 10% for both geometries, showing the validity of the approach independent of the samples’ orientation.

**Table 3:** Corrected Mean Fluorescence Intensities in cps for Confocal Micro-XRF Measurements on Bovine Dentine and SDR Flow+ at A1 First (A) and Second Orientation (B)

	filling–dentine (A)	dentine–filling (B)	relative deviation
Ca Kα	1320	1350	1.1%
Zn Kα	2.8	2.8	0%
Ba Lα	730	660	5%
Yb Lα	300	250	9%
Sr Kα	38	31	5%

**Figure 5:**
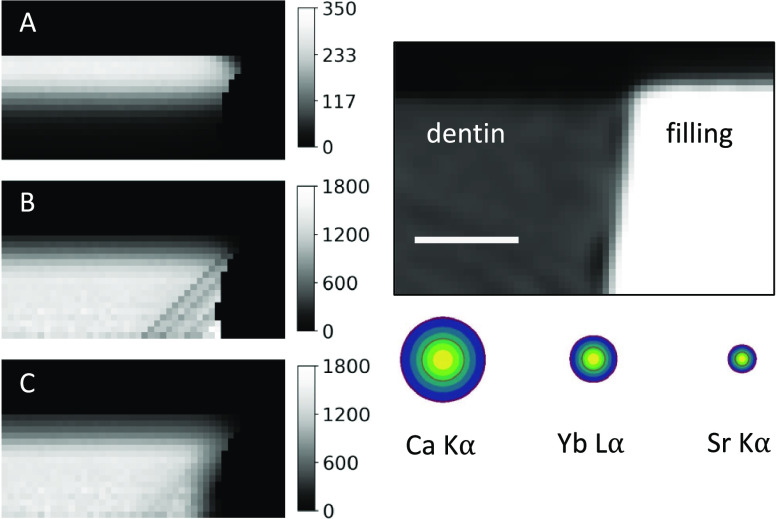
(Left) Corrected elemental distributions of Ca Kα measured at A1. (A) shows the measured elemental distribution. Absorption correction is performed (B) assuming an infinitesimal small probing volume and (C) considering the probing volume as 2D Gaussian distribution. (Right) corresponding micro-CT slice and 2D Gaussian distributions. The scale of the elemental distributions, 2D Gaussian distributions, and the virtual micro-CT slice are the same for easy comparison. The color scale shows the height of the Gaussian distribution. Scale bar: 100 μm.

Consideration of the size of the probing volume is most important for Ca or Ba. As shown in Figure 5 (right), the size of the probing volume is largest for low fluorescence energies (Supporting Information S1). Therefore, in Figure 5 (left), as an example the absorption corrected intensity distributions of Ca Kα assuming the probing volume is an infinitesimally small point (B) and considering the size of the probing volume to be a 2D Gaussian distribution (C) is shown. Artifacts in panel (B) are visible, which are significantly reduced when considering the extension of the probing volume on the right. The higher the fluorescence energy of the elemental distributions, the smaller the influence of the size of the probing volume is.

**Figure 6:**
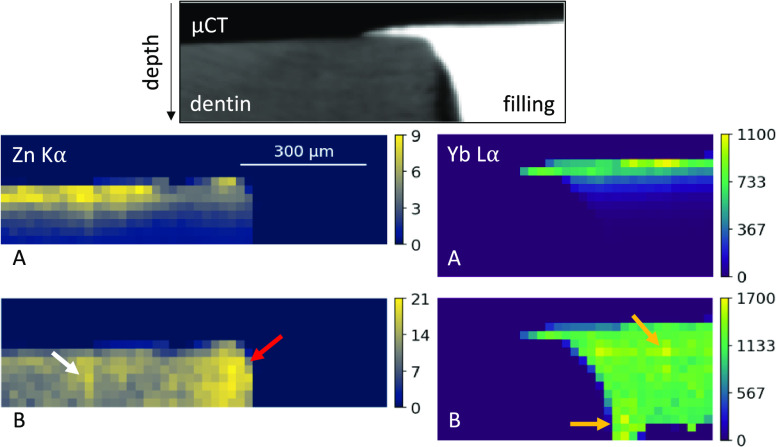
Measured (A) and corrected (B) elemental distributions and corresponding micro-CT virtual slice of partially overlapping interface between filling and dentine (A2). The probing volume size is considered. Color scales show the intensity (cps) of the fluorescence signal. The arrows highlight details in the Zn and Yb distribution. Blue arrow: gap between filling and dentine.

The confocal micro-XRF measurement on A2, which shows a more complex interface, is performed using a step size of 20 μm. Before applying the absorption correction, the confocal micro-XRF data set is scaled using a bicubic interpolation to the approximate same voxel size as the one from the micro-CT data set (6.2 μm). In Figure 6, the rescaled measured and corrected virtual *xz*-slices of the confocal micro-XRF elemental distributions of Yb Lα and Zn Kα measurement as well as the matched micro-CT slice showing the overlapping interface of filling and dentine with a gap underneath the overlapping region.

The corrected distributions make details visible such as a line of higher Zn intensity (white arrow) and a higher Zn signal closer to the root canal (red arrow). Furthermore, small inhomogeneities inside the Yb distribution become visible (orange arrows). The absence of an absorption gradient into the depth of the corrected elemental distributions of Yb and Zn shows that the absorption correction also works for more complex interfaces.

**Figure 7:**
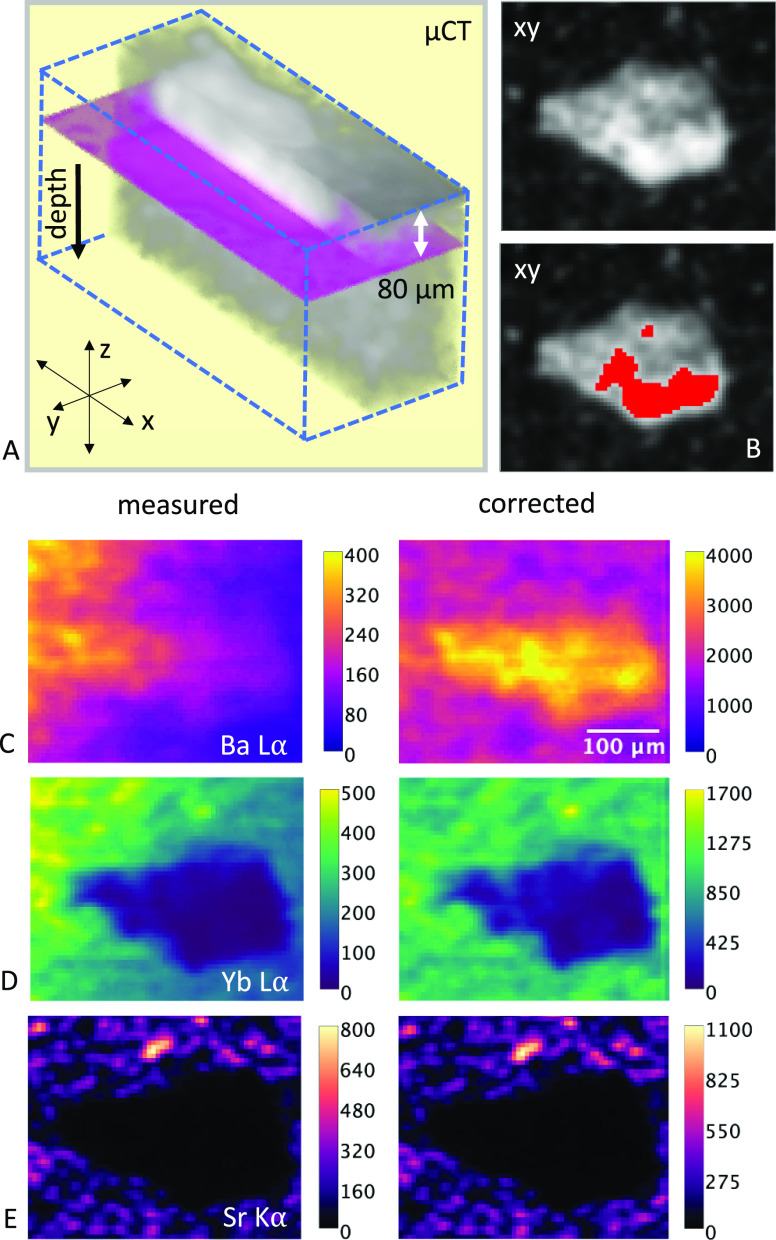
Micro-CT data, measured and corrected elemental distributions of region A3 of the filling with a denser grain. (A) shows a micro-CT volume depicting half of the grain under the surface in the filling material. The magenta-colored slice at a depth of 80 μm marks the virtual slice where the elemental distribution is measured by confocal micro-XRF. (B) shows a virtual micro-CT slice of the grain in *xy*, with and without the more dense area of the grain marked in red. (C–E) show the measured (left) and corrected (right) distributions of Ba, Yb, and Sr. Color scales show the intensity (cps) of the fluorescence signal.

An inclusion grain at A3 is identified with higher density in the micro-CT data set and then measured by confocal micro-XRF. A 3D volume with a voxel size of 18 μm in the xy*z*-directions, which encloses the grain of higher density inside the filling material, is imaged. This 3D data set is scaled using a bicubic interpolation to the voxel size of the micro-CT data set. The coarse measurement is used to match the two data sets and to determine absorption within the grain. The grain is composed of two regions—one containing Ba and Yb with a lower absorption, and the other devoid of Yb yet with a higher absorption. The proposed linear mass absorption coefficients of the grain and the measured and corrected slices of the 3D distribution of Yb are shown in Supporting Information S3 and S4. In addition to the 3D volume, a virtual *xy* slice with a step size of 6 μm is measured representing a virtual cross section of the grain at a depth of approximately 80 μm. At every y position a virtual *xz* slice of the micro-CT measurement is used to calculate the absorption correction. In Figure 7A, a 3D volume of half of the grain and the surrounding filling is depicted, where the position of the virtual *xy* slice measured by confocal micro-XRF is marked in magenta. In Figure 7B, two virtual slices of the micro-CT measurement show the structure of the grain with dimensions of 300 μm × 90 μm × 190 μm at the largest extension. C–E show the measured (top) and corrected fluorescence intensities of Ba Lα, Yb Lα, and Sr Kα. The energy-dependent probing volume size is considered for all three dimensions (*x, y, z*). The performance of the absorption correction is again demonstrated by the corrected elemental distributions which show no gradient inside and outside the grain (as is visible in the measured elemental distributions for Ba Lα and Yb Lα). In Supporting Information S5, the differences in the results applying the effects of the size of the probing volume on the absorption correction are shown.

## Discussion

A detailed voxel-wise absorption correction procedure based on the Lambert–Beer equation of 3D elemental distributions of a heterogeneous composite sample with varying matrix and density is shown. The correction is achieved by combining information from X-ray fluorescence, transmission, and absorption techniques. The results from a geometrically simple interface between filling and dentine shown in Figure 4, and the corrected mean intensity values from the measurements with varying orientation of excitation and detection path, Table 3, show that the absorption correction procedure is suitable for composite samples where the attenuation of different materials close to each other needs to be considered. The measurement shown in Figure 6 demonstrates that the absorption correction works well for complex composite interfaces, uncovering information that was hidden due to absorption effects, as is clearly visible in the Zn distribution. The corrected Zn distribution reveals a higher intensity near the root canal, while 400 μm away from the interface, a line with higher intensity becomes visible. This line is not visible without the absorption correction and corresponds to a Zn-enriched ring around the root canal.

The density information on micro-CT data reveals the general structure of a full sample and delivers important details within the sample, including voids, gaps or grains such as the one shown in Figure 7. While measuring samples with high and low absorbing areas, artifacts, e.g., beam hardening,^30^ need to be considered as visible in Figure 2 (top-right) in stripes emanating from the border between dentine and filling. Here, an orientation was chosen during the measurements to avoid artifacts in the region of interest. Depending on the voxel size, which depends on the setup and the measuring parameters, the size of the detectable structural details is limited. Nevertheless, as long as the voxel size is the same or smaller than the one of the confocal micro-XRF measurement, it yields additional structural information and complements confocal micro-XRF, which is a time-consuming spectroscopic technique.

Compared to micro-XRF, the sensitivity of confocal micro-XRF is reduced due to the transmission of the second polycapillary optic and the small number of excited atoms in the probing volume. Additionally, measurement times are long as the sample is moved in 3D. The combined use of micro-CT information allows for a faster selection of specific sites inside the sample to be measured with confocal micro-XRF without the need for full 3D imaging. It also facilitates the absorption corrections of this data by delivering information such as the length of the excitation and detection paths in different parts of any sample (e.g., tooth tissue and filling).

The importance of considering the probing volume size for larger probing volume sizes combined with high linear mass absorption coefficients needs to be noted. Figure 5 (left) shows the absorption-corrected elemental distributions of Ca with and without considering the probing volume size. The polycapillary optics in the excitation and detection path have a 2D Gaussian-shaped sensitivity distribution. The overlap of the foci forms a rotational ellipsoid with horizontal and vertical dimensions of the FWHM of the Gaussian distributions.^31^ Due to the energy dependency of the FWHM of the focus of the detection lens, the probing volume width is larger for Ca K (3.7 keV, ∼ 50 μm) than Sr K (14.2 keV, ∼ 15 μm). For simpler computation, the probing volume size is approximated as 2D Gaussian distribution, yielding already elemental distributions with fewer artifacts and a more precise absorption correction of the heterogeneous composite samples. As an outlook, the description of the probing volume by an ellipsoid instead of a Gaussian distribution is likely to further improve the absorption procedure.

Besides the need for the correction procedure based on the Lambert–Beer equation, measuring the absorption of the materials of interest in several energy ranges using X-ray absorption and transmission techniques makes an approximation of the mass fractions of the materials possible. Depending on the atomic number and the relative weight percentage of the element, the exact mass fraction has more or less influence on the calculated linear mass absorption coefficient.

Combining the information about the sample composition derived from absorption measurements with the information from micro-XRF intensities and FP quantification of reference-free XRF in the soft energy range yields mass fractions of elements that cannot be obtained with only one technique. If a good guess of the dark matrix is given, e.g., by literature, the analysis of micro-XRF measurements combined with FP calculations can be used to obtain quantitative values for elements above Silicon (1.74 keV). For materials with unknown dark matrix though, such as the filling material in this study, information on the content of dark matrix elements (C, N, and O) derived from, e.g., soft energy range reference-free XRF, is a prerequisite for quantification. Additionally, quantitative values for the concentrations of low-Z elements such as F, Mg, Na, and Al are provided. Absorption inside the sample, which strongly affects the confocal micro-XRF measurements, serves as additional validation of the assumed mass fractions.

In the presented procedure, two constraints need to be considered as crucial for the voxel-wise correction: correct segmentation of the micro-CT data and successful registration of the micro-CT and confocal micro-XRF data. While the latter could be improved experimentally by first measuring a very characteristic structure in 3D for matching purposes only, the former can be tuned within the analysis. Segmentation of the micro-CT data set is needed to separate empty volume fractions, as well as distinct material phases (dentine and filling material), and is required to calculate the respective masks. A binary threshold is used, which makes it challenging to find the surface and the interface simultaneously caused by similar gray values in different phases. As the segmentation is also used to define the number of voxels in the excitation and detection path, an improvement using refined (e.g., deep learning) segmentation methods^32,33^ would make the approach more practicable and adaptable to different kinds of composite samples. It should be mentioned that the necessary accuracy of the mask is, on the one hand, more critical for energies where the linear mass absorption coefficient of the materials within the sample is higher and, on the other hand, where the linear mass absorption coefficient differs substantially between the involved materials. In the tooth filling case, the accuracy of the segmentation of the micro-CT data is most crucial for Ca and Ba and less relevant for Yb, Zn, and Sr.

In this work, nondestructive laboratory and synchrotron experiments are performed to determine the mass absorption coefficient and the elemental composition of the materials as best as possible. Other analytical destructive methods, such as mass spectrometry or chemical analysis, can be used to determine the mean composition if certain experiments are not available, e.g., due to beamtime restrictions. The absorption correction approach can even be applied using a good guess of the sample composition (such as, e.g., collagen + carbonated apatite for dentine). In combination with validation measurements by confocal micro-XRF as presented in Figure 4 and Table 3, significantly improved results are derived when compared to raw data.

## Conclusions

The absorption correction approach presented here reveals details in the elemental composition and structure of composite samples. The example of dental composite materials shows that this approach allows a more precise 3D investigation of diffusion of elements at the interface between tooth and filling, which was hindered by absorption so far.^15^ Furthermore, a voxel-wise quantification of the elemental distributions is also in reach, due to the calculated excitation spectrum in each voxel yielding additional information on the sample matrix and structure. The demonstrated procedure of measuring and calculating the mass absorption coefficient can be applied to other sample materials or combined with other measuring techniques to reveal the elemental composition. With a good initial preknowledge, e.g., on the light elements, the methodology is significantly simplified. Especially, if the mass absorption coefficient is known or measurable, the approach can be adapted to all samples that can be measured by micro-CT and confocal micro-XRF.

## Supporting Information Available

Additional information on the materials and data analysis, and additional measured and corrected elemental distributions (PDF)
