# Supplementary material for: Absorption Correction for 3D Elemental Distributions of Dental Composite Materials Using Laboratory Confocal Micro-X-ray Fluorescence Spectroscopy
Source: Anal Chem. 2024 May 17;96(21):8441–9. doi: 10.1021/acs.analchem.4c00116 (PMC11140690; doi:10.1021/acs.analchem.4c00116)
Supplement: Supplementary file 1 — ac4c00116_si_001.pdf [file ac4c00116_si_001.pdf]

Supporting Information

to

"Absorption correction for 3D elemental distributions of dental composite materials using laboratory confocal micro-X-ray fluorescence spectroscopy"

Leona J. Bauer[1,2,*], Frank Wieder[3], Vinh Truong[1,†], Frank Förste[1], Yannick Wagener[1], Adrian Jonas[4], Sebastian Praetz[1], Christopher Schlesiger[1], Andreas Kupsch[3], Bernd R. Müller[3], Birgit Kanngießer[1], Paul Zaslansky[5], Ioanna Mantouvalou[2]

1) *Institute for Optics and Atomic Physics, Technical University of Berlin, Hardenbergstr. 36, 10623 Berlin, Germany; Berlin Laboratory for innovative X-ray technologies – BLiX; 2) Helmholtz Zentrum Berlin, Albert-Einstein-Str. 15, 12489 Berlin, Germany; 3) Bundesanstalt für Materialforschung und -prüfung (BAM), Unter den Eichen 87, 12205 Berlin, Germany; 4) Physikalisch-Technische Bundesanstalt, Abbestraße 2-12, 10587 Berlin, Germany; 5) Department for Operative, Preventive and Pediatric Dentistry, Charité - Universitätsmedizin Berlin, Aßmannshauser Str. 4-6, 14197 Berlin*

*Corresponding author: leona.j.bauer@campus.tu-berlin.de

<u>Table of content</u>

S1 Materials & Methods
- S1.1 Materials – Preparation of teeth and dental materials
- S1.2 FWHM of the used CµXRF setup

S2 Methodology
- S2.1 Determination of the effective *µl* in the excitation path
- S2.2 Registration of µCT & CµXRF data to determine absorption paths

S3 Results
- S3.1 Forward calculated and measured fluorescence intensities
- S3.2 Measured and corrected fluorescence intensities at A1 second orientation
- S3.3 Linear mass absorption coefficients defined for the two phases of the grain
- S3.4 measured and corrected fluorescence intensities of the measurement at A3
- S3.5 Comparison of absorption corrected values in an identified grain with and without considering the probing volume size

References

S1 Materials & Methods

S1.1 Materials – Preparation of teeth and dental materials

The teeth were extracted from the jaws of 3–8 year-old slaughtered bovines, which were provided by a butcher (*Henke Qualitätsfleisch, Germany*). After extraction, the teeth were stored in a Chloramine-T solution to prevent bacterial and fungal infection.

One tooth was measured by µCT wet untreated and is shown for visualization purposes (Figure 2, top-left). The root region from a second bovine tooth was cut using a water-cooled diamond saw (*Exakt, Germany*) into a 3 mm thick cross-section. The root canal of this cross-section was then filled with a dental biomaterial: SDR flow+ (Figure 2, top-right) using a bonding agent commonly used to attach composites to dentine (Futurabond U, VOCO GmbH, Germany). Both materials were light-cured with a clinically used LED dental source. This sample was then used for quantification using ~ 850 µm thick slices produced by a water-cooled diamond saw. (sample marked T1, Figure 2, bottom). This thickness is most suitable for sample measurements in the range of 17-23 keV.

A second sample was prepared by cutting and polishing to 144 µm in cross-section for transmission measurements at energies in the range of 3 keV - 10 keV (sample marked T2). For composite measurement of transmission and absorption, two planar disks were flattened and light-cured yielding 2 samples with thicknesses of 123 µm (named SDR-thin) and 413 µm (named SDR-thick) and a diameter of 8 mm.

S1.2 FWHM of the used CµXRF setup

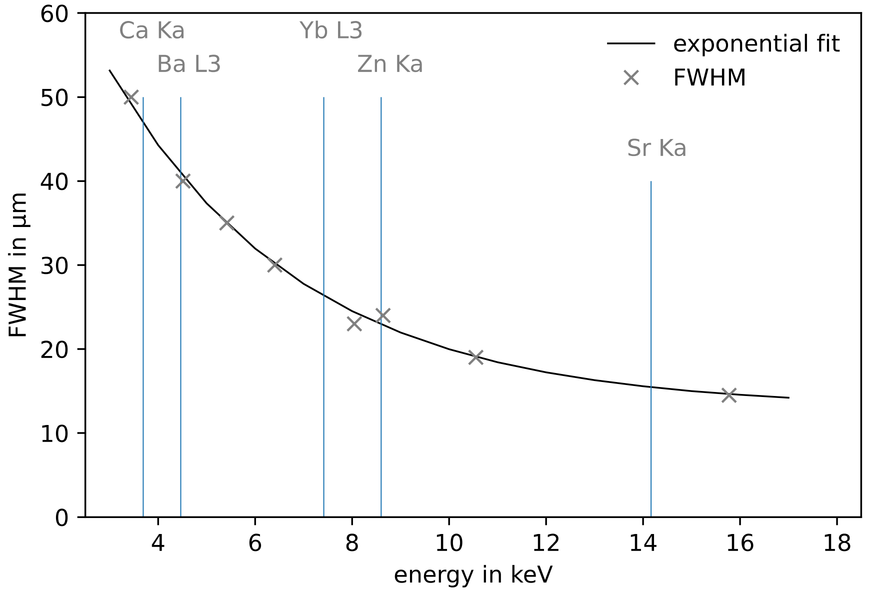

Figure S1: Depth resolution of the CµXRF setup. FWHM values were derived from measurements of thin metal foils, 5 µm step size, 10 s measurement per spectrum. The exponential fit is used to derive FWHM values for the line energies of the elements needed for the absorption correction. These are indicated with vertical blue lines.

S2 Methodology

S2.1 Determination of the effective *µl* in the excitation path

An "effective excitation energy" approximation [1,2] is used for the absorption correction procedure. This effective energy is then used to find the effective linear absorption coefficient per depth (*µl)* of each voxel (*x,y,z*). Unlike previous approaches, where a single depth scan on a homogeneous sample can be used to determine the effective excitation energy at different depths, here effective energies must be calculated for each voxel separately. This is because absorption depends not only on the depth and matrix of a material but also on the path that the incident radiation propagates through along the different materials of the inhomogeneous sample. We use a calculated excitation spectrum for each voxel – taking into consideration the attenuation of the initial spectrum within the sample. The excitation spectrum of the Mo microfocus X-ray source is calculated using the Elam database [3]. This spectrum is multiplied by the Gumbel function to approximate transmission of the lens focusing the incoming X-rays. The Gumbel function is derived from µXRF point measurements on single-element reference samples [4,5]. The attenuation of the spectrum within the sample is then calculated using a linear combination of the linear mass absorption coefficients of the materials with the Lambert-Beer function.

The product of the effective absorption coefficients and the thickness μl in each voxel (x,y,z) is calculated by dividing the initial excitation spectrum I_0 by the attenuated spectrum I_{atten} at the effective excitation energy E_{eff} of a given element line *j* according to the Lambert-Beer equation:

$$\mu d(E_{eff,j},x,y,z) = -\ln\left(\frac{I_{atten}(E_{eff,j},x,y,z)}{I_0(E_{eff,j})}\right). \quad (1)$$

S2.2 Registration of µCT & CµXRF data to determine absorption paths

In order to use the structural information provided by the µCT measurement for the voxel-wise absorption correction of the CµXRF data, the two data sets must be matched for which we used an image registration approach. For this purpose, the CµXRF measurements were scaled to fit the µCT measurement using bicubic interpolation. The µCT data and the CµXRF data are aligned in 2 rotational and 3 translational directions (*x,y,z*) with a template matching algorithm available in python (module: *cv2*, function: *matchTemplate,* attribute: *TM_SQDIFF_NORMED).* Within this algorithm, the template image (CµXRF data) is rotated in 1-degree steps and translated pixel-wise through the source image (µCT data set) to find best match.
As the CµXRF data set is very small compared to the µCT data, we helped the alignment by pre-matching the data using a 2D µXRF map measured under the same sample setup geometry. The pre-aligned µCT data is then used to register the CµXRF data.

In order to calculate the thicknesses of the different materials in the excitation and detection paths of the CµXRF measurement, density information derived from the µCT data is used. Binary thresholds were defined to classify the voxels according to the different materials, also identifying areas where the probing volume was not inside the sample. At each position within the sample, the number of voxels considered as belonging to the sample is summed over all preceding voxels within the excitation and detection paths. Note that we use excitation and detection angles of 45°. Consequently we end up with a table listing the number of voxels in the excitation and detection paths, separated according to the materials with different absorption coefficients, for each measurement point in the sample.

S3 Results

S3.1 Forward calculated and measured fluorescence intensities

Table S1: Calculated and measured fluorescence intensities in cps for µXRF measurements on bovine dentine and SDR flow+ dental composite. Similar deviations were estimated from additional measurements and calculations on multielement reference glass samples (Breitländer GmbH).

material	element line	measured	calculated	deviation
dentin	P Kα1 + Kα2	96	124	28 (30 %)
	Ca Kα1 + Kα2	4050	4860	810 (20 %)
	Zn Kα1 + Kα2	40	40	0 (0 %)
	Sr Kα1 + Kα2	50	54	4 (8 %)
filling	Ba Lα1 + L Kα2	1870	1430	440 (24 %)
	Yb Lα1 + Lα2 + Lβ + Ll	2630	2900	270 (10 %)
	Sr Kα1 + Kα2	4180	4000	180 (5 %)

S3.2 Measured and corrected fluorescence intensities at A1 second orientation

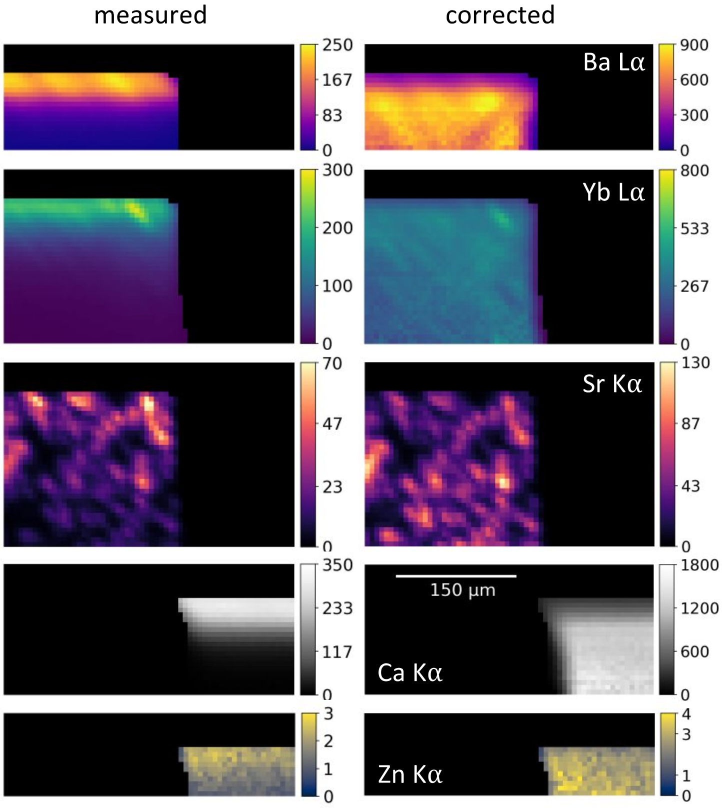

Figure S2: Measured and corrected elemental distributions of Ba, Yb, Sr, Ca, and Zn at A1 second orientation. Color scales show the intensity (CPS) of the fluorescence line.

S3.3 Linear mass absorption coefficients defined for the two phases of the grain

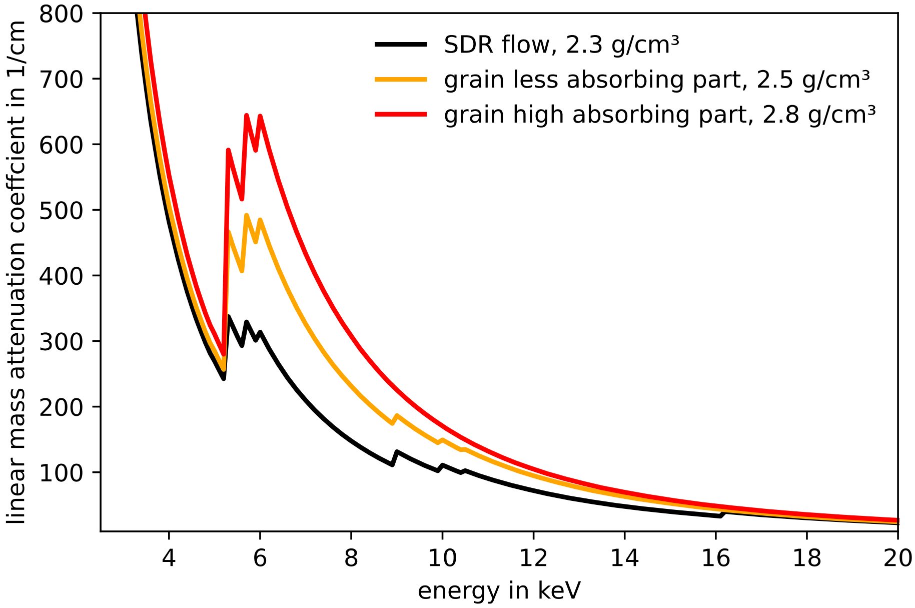

Figure S3: Linear mass absorption coefficients for the filling material and two regions of a grain identified inside the filling. The composition of the grain was assumed to have the same composition as the filling but lacking Sr and containing different amounts of Ba and Yb. Two different composition and density regions were observed in the grain, identified using coarse 3D measurement around the grain (see Figure S4). From the absorption corrected elemental distribution below the grain, the composition and density were adapted to fit the absorption coefficient at the energy of Ba Lα, Yb Lα, and Sr Kα. One part of the grain is thus assumed to contain Ba and Yb but no Sr (orange, density 2.5 g/cm3). The second part of the grain seemingly contains only Ba (red, density 2.8 g/cm3).

S3.4 measured and corrected fluorescence intensities of the measurement at A3

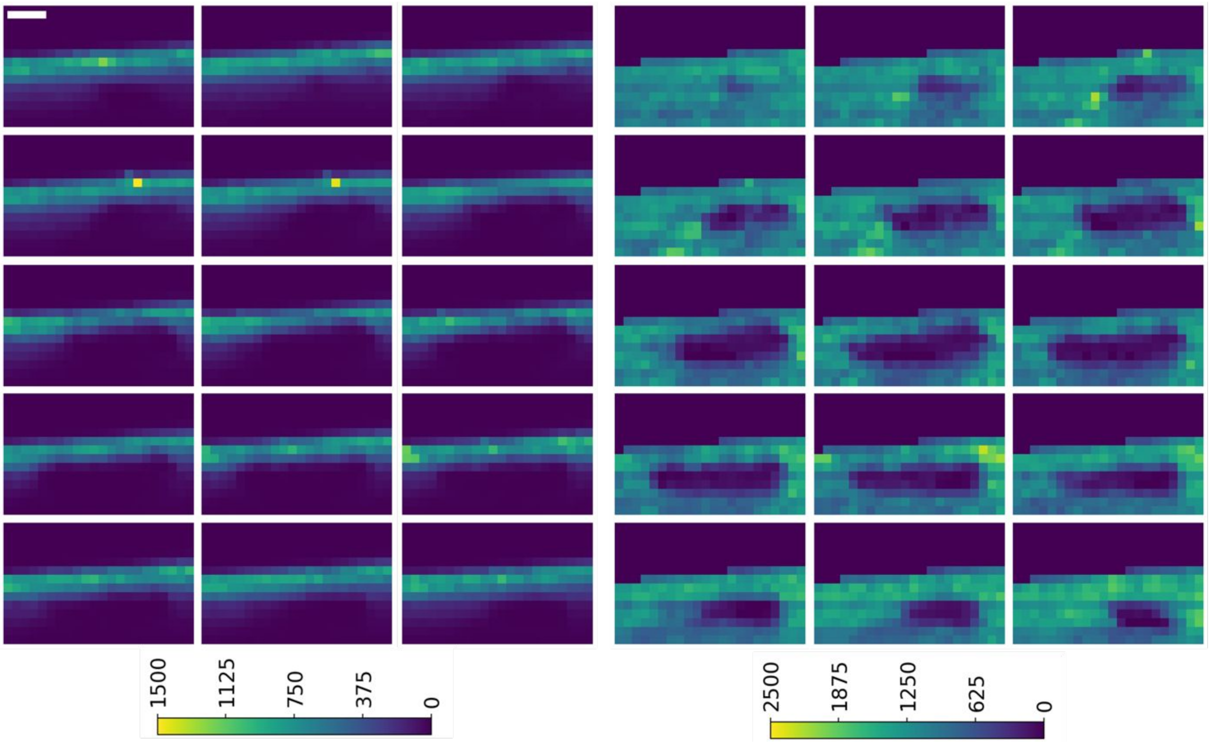

Figure S4: Measured (left) and corrected (right) elemental Yb Lα distribution at A3 with a step size of 18 µm x 18 µm x 18 µm. Only every second virtual xz slice of the 3D measurement is shown. Color scales show the intensity (CPS) of the fluorescence. Scale bar: 100 µm.

S3.5 Comparison of absorption corrected values in an identified grain with and without considering the probing volume size

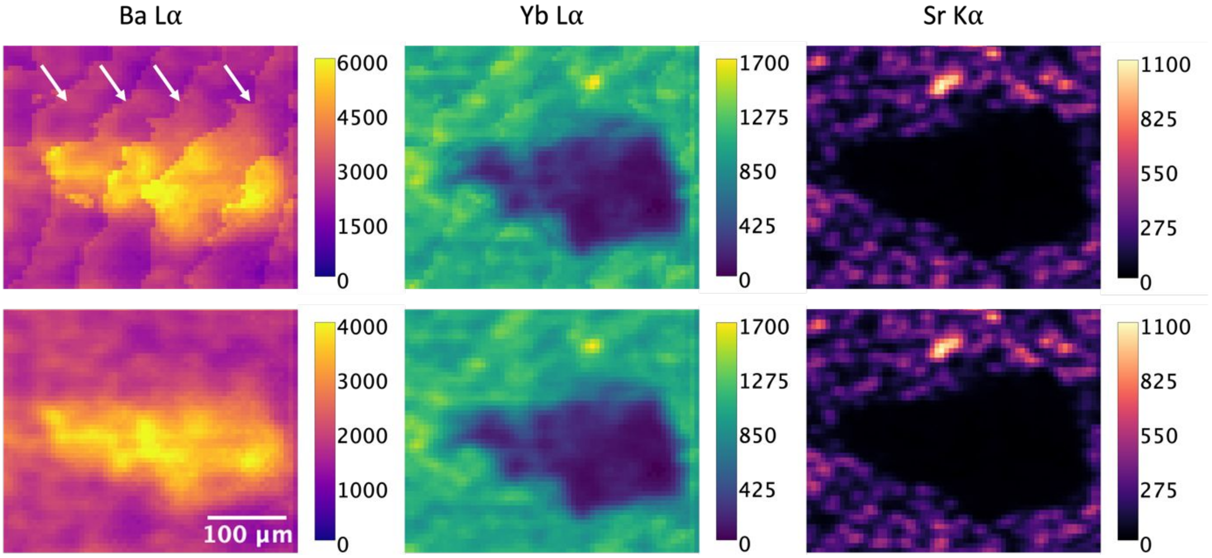

Figure S5: Corrected elemental distributions of Ba, Yb, and Sr at A3 with a step size of 6 µm x 6 µm, results obtained with 2 different approaches. (top) assuming an infinitesimal small probing volume size, (bottom) considering a 3D Gaussian distributed probing volume size. Color scales show the intensity (CPS) of the fluorescence. The Ba distribution corrected for an infinitesimal small probing volume shows plateau-like artifacts (white arrows) identified the surface of the sample mask.

References:

1. Mantouvalou, I.; Lachmann, T.; Singh, S. P.; Vogel-Mikuš; K.; Kanngießer, B. Advanced Absorption Correction for 3D Elemental Images Applied to the Analysis of Pearl Millet Seeds Obtained with a Laboratory Confocal Micro X-ray Fluorescence Spectrometer. *Anal. Chem.* **2017**, *89*, 5453–5460.
2. Wrobel, P.; Wegrzynek, D.; Czyzycki, M.; Lankosz, M. Depth profiling of element concentrations in stratified materials by confocal microbeam X-ray fluorescence spectrometry with polychromatic excitation. *Anal. Chem.* **2014**, *86*, 11275–11280.
3. Elam, W. T.; Ravel, B. D.; Sieber, J. R. A new atomic database for X-ray spectroscopic calculations. *Radiat. Phys. Chem.* **2002**, *63*, 121–128.
4. Förste, F.; Bauer, L.; Heimler, K.; Hansel, B.; Vogt, C.; Kanngießer, B.; Mantouvalou, I. Quantification routines for full 3D elemental distributions of homogeneous and layered samples obtained with laboratory confocal micro XRF spectrometers. *J. Anal. At. Spectrom.* **2022**, *37*, 1687–1695.
5. Bauer, L. J.; Gnewkow, R.; Förste, F.; Grötzsch, D.; Bjeoumikhova, S.; Kanngießer, B.; Mantouvalou, I. Increasing the sensitivity of micro X-ray fluorescence spectroscopy through an optimized adaptation of polycapillary lenses to a liquid metal jet source. *J. Anal. At. Spectrom.* **2021**, *36*, 2519–2527.
